# Multiple Mating of *Aphelinus asychis* Enhance the Number of Female Progeny but Shorten the Longevity

**DOI:** 10.3390/insects12090823

**Published:** 2021-09-14

**Authors:** Shengyin Wang, Libo Wang, Jiawen Liu, Dayu Zhang, Tongxian Liu

**Affiliations:** 1 College of Advanced Agricultural Sciences, Zhejiang A&F University, Hangzhou 311300, China; 20160040@zafu.edu.cn (S.W.); liujiawen46@163.com (J.L.); zhangdayu@zafu.edu.cn (D.Z.); 2 College of Economic and Management, Zhejiang A&F University, Hangzhou 311300, China; zafuwlb@163.com; 3 College of Plant Health and Medicine, Qingdao Agricultural University, Qingdao 266109, China

**Keywords:** biological control, green peach aphid, mating behaviour, backcross, control efficiency

## Abstract

**Simple Summary:**

*Aphelinus asychis* Walker is an arrhenotocia endoparasitoid against the devastating vegetable pest *Myzus persicae.* Unmated *Aphelinus asychis* females only produce male progeny, and mated female adults produce male and female progeny. Because only female adults can kill the target pest by parasitism and feeding, the control efficiency of *Aphelinus asychis* was mainly affected by the percentage of female adults. We found that *Aphelinus asychis* females could mate multiple times to receive more sperm in their life span, which was beneficial for enhancing the number and percentage of female progeny. In addition, backcrossing is critical for population increase when the proportion of males is low. We also found that there was no significant difference in the population fitness of *Aphelinus asychis* between backcross and control treatments.

**Abstract:**

The *Aphelinus asychis* female adult is an important arrhenotocous parthenogenesis parasitoid of *Myzus persicae*, and its reproductive mode is beneficial for the population continuation of *A. asychis* by way of multiple mating and backcross. To explore the effect of mating on the population fitness and control efficiency of *A. asychis*, its mating frequency and backcross were observed under laboratory conditions. The results showed that most matings in *A. asychis* involved four distinct stages: courtship, pre-copulatory, copulation, and post-copulatory behaviours. Only the duration of courtship increased significantly with an increase in copulation frequency for females, and the courtship duration of *A. asychis* females mated with different males were significantly shorter than those mated with the same male at the same mating times, which suggested that *A. asychis* females might prefer to mate with different males to enrich the genotype of their offspring. The total number of mummified aphids and the female and male longevity decreased significantly with an increase in mating frequency. On the contrary, female progenies increased significantly with an increase of mating frequency, suggesting that sperm limitation might occur in females when they only mated once. These results imply that females might prefer to receive more sperm by mating multiple times in their life span. In addition, we found that the intrinsic rate of increase (*r*) of *A. asychis* of the control group (0.2858 d^−1^) was significantly greater than that in the backcross treatment (0.2687 d^−1^). The finite killing rate (*θ*) of *A. asychis* of the control group was similar to that in the backcross treatment, which showed that this treatment had a negligible negative effect on the control efficiency of *A. asychis*. In conclusion, the results showed that multiple mating increased the number and proportion of *A. asychis* female progenies but shortened the longevity of female and male adults, while the negative effect of backcross on the control efficiency of *A. asychis* was negligible.

## 1. Introduction

*Aphelinus asychis* Walker (Hymenoptera: Aphelinidae) is a polyphagous and arrhenotocous endoparasitoid native to Europe, Asia, and Africa [1,2]. This species is capable of parasitizing about 40 aphid species, especially for *Myzus persicae* Sulzer [3,4], which infests the chili pepper in the greenhouse and field [4,5]. In addition, host feeding by *A. asychis* females also causes mortality to *M. persicae* and plays a crucial role in the egg maturation of synovigenic parasitoids [6,7]. *A. asychis* has been considered as a potential candidate for the biological control of *M. persicae* [3,4,5,6]. According to the previous research, the control efficiency, including parasitism and host feeding, of *A. asychis* is significantly affected by environmental temperature [4,6].

Because insects are ectotherms, their behavior and fitness are affected by the ambient temperature, particularly abnormally high or low temperatures, and any changes in environmental temperature may impact their fitness and life history traits, and thus, affect population dynamics [8,9]. Therefore, the influence of temperature on parasitoids has been studied for such species as *Spalangia cameroni* and *Muscidifurax raptorellus* [10], *Aphidius avenae* [11], *Trichogramma poliae* and *Trichogramma chilonis* [12], and *Trichogramma evanescens* [13]. Generally, the temperatures under greenhouse conditions show irregular cyclic variation daily, and midday temperatures often exceeded 40 °C for a period of time [14]. In addition, global warming has become a social consensus, and the impact of global warming on agricultural pests and their natural enemies has been researched for many years [10]. In previous research, Wang et al. found that *A. asychis* females were more tolerant to high temperature than males at 40 °C, and the proportion and longevity of *A. asychis* males decreased significantly because of their high mortality under high-temperature environmental conditions [5]. Therefore, a suitable proportion of *A. asychis* males are necessary for the maintenance of population balance or to increase their population in an extreme high-temperature environment, which may be beneficial to increase the control efficiency. Thus, backcross is critical for population increase when the number of *A. asychis* males is low. However, backcross might lead to exposure to defects or lethal genes, which is disadvantageous to the continuity of the population [15]. Therefore, the effect of backcross on the population fitness of *A. asychis* is important and should be researched.

Multiple mating is a widespread phenomenon in various insect species [16,17] which provides more mating opportunities for males and increases female fecundity [17]. For *Spalangia endius* Walker (Hymenoptera: Pteromalidae), female adults mate only once, but males can mate with many females [18,19,20,21], which is beneficial for male adults propagating its sperm to females as much as possible. However, an increase in the frequency of mating for males results in a gradual decrease in the amount of sperm supplied to females at each mating [20], which could affect female reproduction by way of less sperm and less nutrition [19,20,21,22]. Generally, unfertilized and fertilized eggs develop into male and female adults, respectively [23]. Female adults that lack sperm would produce more unfertilized eggs and increase the percentage of male progenies [24,25]. Therefore, the proportion of the next generation *A. asychis* females may be affected by mating times, which may be used to infer the control efficiency of *A. asychis* females in the next generation.

In previous studies, only the *A. asychis* female was shown to be capable of parasitizing and feeding on *M. persicae* [3,4,6,26], and only mated females are able to produce female progeny [1]. Therefore, an appropriate proportion of *A. asychis* males are beneficial to ensure the persistence of the population and to enhance the control efficiency of the target pest. To enlarge *A. asychis* populations under unsuitable conditions, in-depth knowledge on the reproduction of *A. asychis* is important. In particular, this study describes the mating behavior and effect of multiple matings on longevity, fecundity, and host feeding. In addition, the influence of backcross on the population fitness and control efficiency of *A. asychis* was evaluated by the two-sex life-table method.

## 2. Materials and Methods

### 2.1. Insects and Plant Cultures

*Myzus persicae* and *A. asychis* were originally collected from the greenhouses of the Key Laboratory of Applied Entomology, Northwest A&F University (Yangling, Shaanxi, China) in 2013 and maintained in air conditioned insectaries [25 ± 0.5 °C, 70 ± 5% RH, with a photoperiod of 14:10 (L:D) h]. The parasitoid *A. asychis* was reared with *M. persicae* that seriously damages chili pepper plants (*Capsicum annuum* L., var. Ox horn) as host [4,5]. The chili pepper plants (~90 days old) were grown in 12 cm diameter plastic pots filled with soil mix (peat moss: perlite = 3:1) and enclosed in nylon net cages (60 × 60 × 60 cm^3^). The plants were watered and fertilized (Compost, COMPO Expert GmbH, Münster-Handorf, Germany) as needed [6].

### 2.2. Mating Behaviour Assay

A mating behaviour assay was conducted using a pair of newly emerged adult female and male (<24 h old) captured and allowed to mate in 250 μL centrifuge tubes. The duration and phase of mating were recorded. Data on mating that lasted more than 1 h were excluded from analysis, because there was no mating behavior for most of those parasitoids even if the duration extended to 5 h. The mating behaviour of same *A. asychis* female adult with the same male adult or different virgin male adults was observed daily. After mating, the female and male were separately reared in a Petri dish which contained a leaf disc and 50 2nd instar nymphs of *M. persicae*. The mating assay of a female adult mating with the same male adult for 1–5 times was repeated for 40, 37, 32, 25, and 15 times, respectively, and the mating assay of female adult mating with the different male adults for 1–5 times was repeated for 40, 34, 28, 19, and 12 times, respectively. Because not all males completed five mating times, the number of repeat times decreased with an increase in mating times. The virgin males without mating were used as controls.

### 2.3. Influence of the Mating Frequency on the Population Fitness of A. asychis

Second instar *M. persicae* nymphs (50) were obtained following the method of Wang et al. [4]. Three milliliters of water-agar (1%) was trickled into a Petri dish (3 cm diameter). After refrigeration, the leaf discs (3 cm diameter) of chili pepper were individually placed on the agar gel surface in the Petri dish. Then, 30 *M. persicae* adults reared on chili pepper plant were placed into each Petri dish to feed and reproduce. After 24 h, those aphid adults were removed, and the newborn nymphs remained on the leaf disc for another 24 h. Newly molted second instar nymphs (50) were used in the experiments, and all younger nymphs and the ecdyses were removed. *A. asychis* female adults mated once and were allowed to oviposit between matings daily. The *A. asychis* female adults that had mated 1, 2, 3, 4, or 5 times were placed in a Petri dish which contained 50 2nd-instar nymphs for feeding or parasitism daily, until the female adults died. According to previous research, the number of *M. persicae* nymph was enough for daily parasitism and host feeding of *A. asychis* [4,6]. After recording the number of aphids fed on by the parasitoid female adults, the Petri dishes containing *M. persicae* nymphs were kept in an incubator at 25 ± 0.5 °C, 70 ± 5% RH and a photoperiod of 14:10 (L:D) until unparasitized nymphs developed into adults or parasitized nymphs mummified. The longevity of *A. asychis* female and male adults, number and emergence rate of mummified aphids, proportion of *A. asychis* female progeny, and the number of *M. persicae* nymphs that was killed for host feeding were recorded. The mating assay with the same or different male adults was repeated 10 times, and virgin females and males without mating were used as controls.

### 2.4. Influence of Backcross on the Population Fitness of A. asychis

The effect of backcross on the population, host feeding, non-effective parasitism and control efficiency of *A. asychis* to *M. persicae* was measured following the method of Wang et al. [4,6]. Fifty mummies in the backcross experiment were produced by ten *A. asychis* female adults after mating with their own male progeny. In the control group, the same amount of mummies was produced when ten females mated with other males. The egg and larval stages were regarded as the first developmental stage. The change in colour of mummified aphid to black indicated the *A. asychis* larvae had pupated [4,5,6]. In the backcross treatment, newly emerged *A. asychis* female and male adults which came from the same female adult were paired. If the male parasitoid died before the female, it was replaced with male adults from the same female. In control, newly emerged *A. asychis* female and male adults from different female adults were paired. If the male parasitoid was not accepted or died before the female, it was replaced with male adults from the other population. The backcross treatments and control were repeated 50 times, respectively. Each pair of *A. asychis* adults was reared in a Petri dish containing 50 2nd instar *M. persicae* nymphs, and transferred into a new one 24 h later. The parasitized and healthy aphid nymphs were maintained in the greenhouse at 25 °C. The developmental time, longevity, parasitism, non-parasitism, and host feeding of *A. asychis* were recorded until all the tested parasitoids died.

The raw data on the population fitness (effective parasitism) of *A. asychis* female individuals were analyzed using the computer program TWOSEX-MSChart [27]. Data on daily host feeding rate, non-effective parasitism rate, host feeding, and aphid killing rate were analyzed following the method of Wang et al. and the computer program CONSUME-MSChart [28]. The parameter definitions and formulas used in this study are presented in Table 1.

### 2.5. Statistical Analysis

The duration of mating stage was analyzed by the Tukey-B multiple comparison test (*p* < 0.05) and *t*-test (*p* < 0.05) using the SAS 2003 software (SAS Institute, Cary, NC, USA). The longevity of *A. asychis* females and males, the number of mummified aphids, and *A. asychis* female progenies from the assays of females which mated with the same or different males were analyzed with the same method. To satisfy the condition of homogeneity of variance, before the one-way ANOVA (SAS Institute, 2003) were performed, all percentage data (e.g., emergence rate of mummified aphids and percentage of female progeny) were square-root transformed, although untransformed results have been presented in all figures. The means, variances, and standard errors of the population parameters, host feeding, non–effective parasitism, and aphid killing were estimated with the bootstrap technique. Because bootstrapping uses random resampling, a small number of replications will generate variable means and standard errors [29,30]. To generate less-variable results, we used 100,000 replications in this study.

## 3. Result

### 3.1. Effect of Mating Times on the Female Fitness

#### 3.1.1. Observation of Mating Behavior and Duration

The mating behaviors of the parasitoids were divided into two types. The first type included four stages: (1) courtship, (2) pre-copulatory behaviour, (3) short time copulation, and (4) post-copulatory behaviour. The second type included only three stages: (1) courtship, (2) pre-copulatory behaviour, and (3) a long duration of copulation. In the courtship stage, *A. asychis* males used their antennae to touch the females’ bodies quickly. At the second stage, *A. asychis* males spread their wings and tried to climb onto the females’ backs. At the third stage, *A. asychis* males climbed onto the females’ backs to complete mating. In the forth stage, *A. asychis* males used the antennae to soothe females and spread their wings to tidy up the whole body. The second type is extremely rare (<5%), so we only analyzed the first type.

In the mating duration treatments, the same female mated with either same single males or different males. For the same single male, the courtship duration increased significantly with an increase in mating frequency (df = 4, 128; *F* = 76.509; *p* < 0.001). However, the pre-copulatory behaviour, copulation, and post-copulatory behaviour durations varied. For different males mated with the same female, the courtship duration also extended significantly as the frequency of mating increased (df = 4, 128; *F* = 60.195; *p* < 0.001), but the duration in other stages varied. Except for the first time mating, the duration of courtship in the same male treatments was significantly longer than that in the different males’ treatment at the same mating frequency (2 time: *F* = 9.527, *p* < 0.001; 3 times: *F* = 7.350, *p* < 0.001; 4 times: *F* = 6.331, *p* < 0.001; 5 times: *F* = 6.819, *p* < 0.001). There were no significant differences in the durations of pre-copulatory, copulation, and post-copulatory behavior at the same mating frequency (Table 2).

#### 3.1.2. Longevity

**Figure 1:**
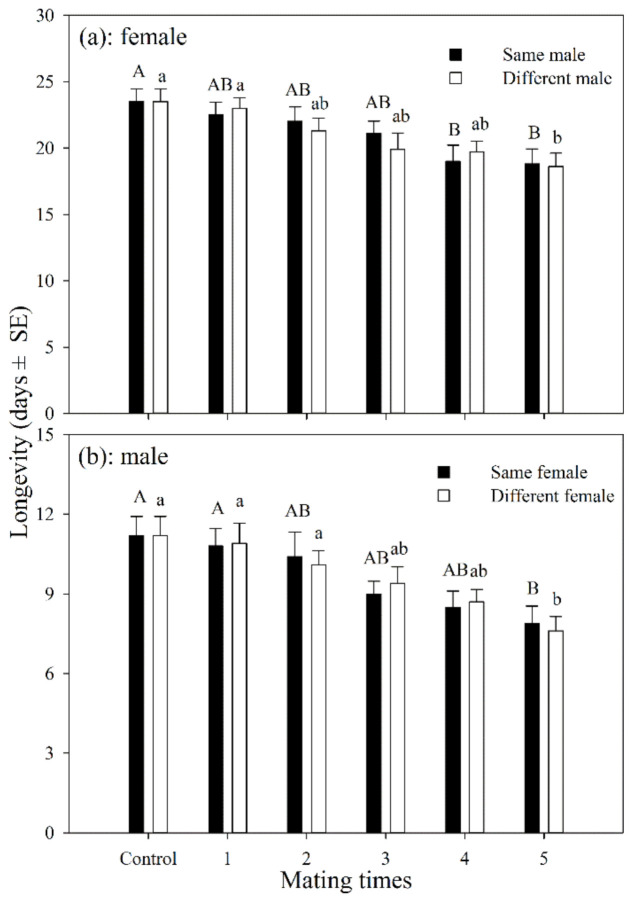
Longevity of *A. asychis* female (**a**) and male (**b**) which mated with the same or different partners. Different uppercase and lowercase letters represent significant differences among multiple mating to same or different partners, respectively (Tukey-B, *p* < 0.05).

The longevity of female adults that had mated with the same male 1–5 times was shorter than those in the control group, but was only significantly different after having mated 4 and 5 times (df = 5, 54; *F* = 3.249; *p* = 0.012). The longevity of mated female adults was shorter than those in the control group, but was only significantly different for those that had mated 5 times (df = 5, 54; *F* = 3.946; *p* = 0.004). There was no significant difference in longevity between females that mated with the same male or with multiple males at the same mating frequency (Figure 1a).

The average longevity of male adults that mated with the same female for 1–5 times was shorter than those in the control group, and was only significantly different for those that mated 5 times (df = 5, 54; *F* = 3.882; *p* = 0.004). The average longevity of male adults that mated with different females 1–5 times was also shorter than the control group, but was only significantly different for those that mated 5 times (df = 5, 54; *F* = 4.935; *p* = 0.001). There was no significant difference in longevity between the males that mated with the same female or with multiple females at the same mating frequency (Figure 1b).

#### 3.1.3. Mummified Aphids and Female Progenies

**Figure 2:**
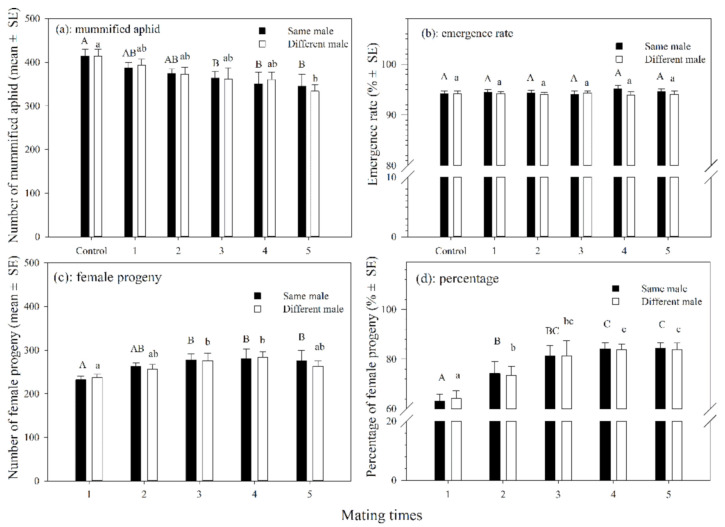
The number (**a**) and emergence rate (**b**) of mummified aphids and number (**c**) and percentage (**d**) of female progenies produced by *A.* *asychis* which mated with the same or different males. Different uppercase and lowercase letters represent significant differences, respectively (Tukey-B, *p* < 0.05).

The number of mummified aphids produced by female adults that mated with the same single male 3–5 times was significantly less than those in the control (df = 5, 54; *F* = 11.027; *p* < 0.05). The number of mummified aphids produced by female adults that mated with different males 1–5 times was also not significantly different from those in the control group except for those that mated 5 times, which significantly differed from those in the control (df = 5, 54; *F* = 2.442; *p* = 0.046). There was no significant difference in the number of mummified aphids between female parasitoids that mated with the same males or different males at the same mating frequency (Figure 2a). The emergence rates of parasitoids that mated with the same male 1–5 times or those that mated with different males varied slightly, but were not significantly different from those in the control group (Figure 2b).

The number of female progenies increased significantly as the frequency of mating increased in both treatments in which the female mated with the same or different males. In addition, there was no significant difference in the number of female progenies between the female parasitoids that mated with the same males and with different males at the same mating frequency (Figure 2c). The percentage of female progenies increased significantly from 63.2% to 84.5% (df = 5, 54; *F* = 67.159; *p* < 0.001) for females that mated with the same male for 1–5 times. The percentage of female progenies varied from 64.1% to 83.9% (df = 5, 54; *F* = 49.846; *p* < 0.001) for females that mated with different males. In addition, there was no significant difference in the percentage of female progenies between females that mated with the same male or different males at the same mating frequency (Figure 2d).

#### 3.1.4. Host Feeding

**Figure 3:**
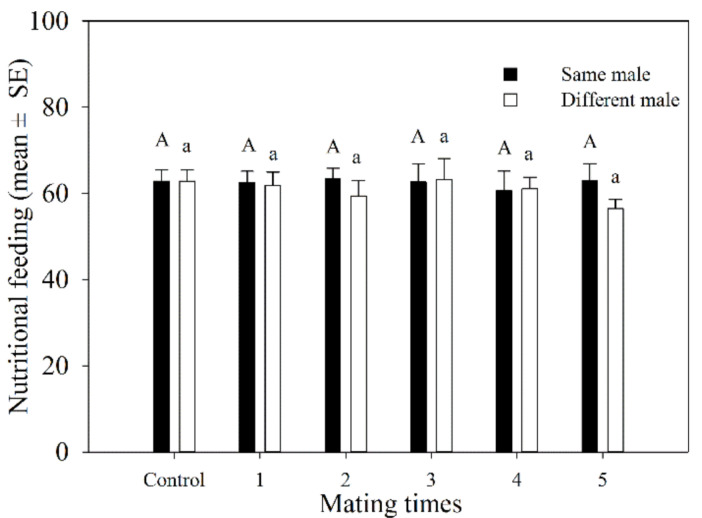
Host feeding number of *A. asychis* females which mated with the same and different males. Different uppercase and lowercase letters represent significant differences among multiple matings to same or different partners, respectively (Tukey-B, *p* < 0.05).

The total number of aphids killed by female adults during mating 1–5 times with the same male was similar. It ranged from 60.6 to 63.5. The total number of hosts that were fed on by females that mated with different females 1–5 times varied from 56.5 to 63.2. There was no significant difference in the total number of hosts fed on by *A. asychis* females between those that mated with the same or different males at the same mating frequency (Figure 3).

### 3.2. Backcross Experiment

#### 3.2.1. Age-Stage, Two-Sex Life Table

**Figure 4:**
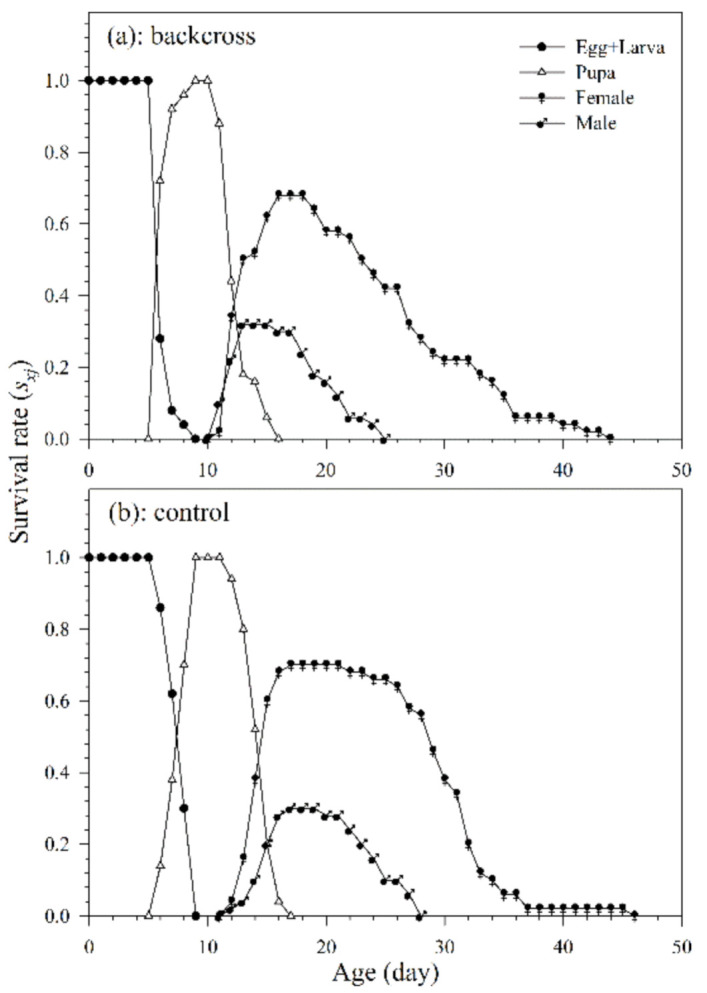
The age-stage specific survival rates (*s_xj_*) of *A. asychis* in the backcross treatment (**a**) and control (**b**).

Of the 50 mummified aphids collected, 35 females of *A. asychis* from the backcross treatment and 34 females from the control group pupated and successfully emerged. The variable developmental rates among individuals, as shown in the age-stage survival curve, are depicted as the overlaps between different stages during developmental periods (Figure 4). The developmental duration of *A. asychis* female and male in the backcross treatment was significantly longer than those in the control. The longevity of *A. asychis* females and males in the backcross treatment and control showed a similar trend. In addition, there was no significant difference in both the oviposition period and average number of progeny adults per female between backcross treatment and control (Table 3).

**Figure 5:**
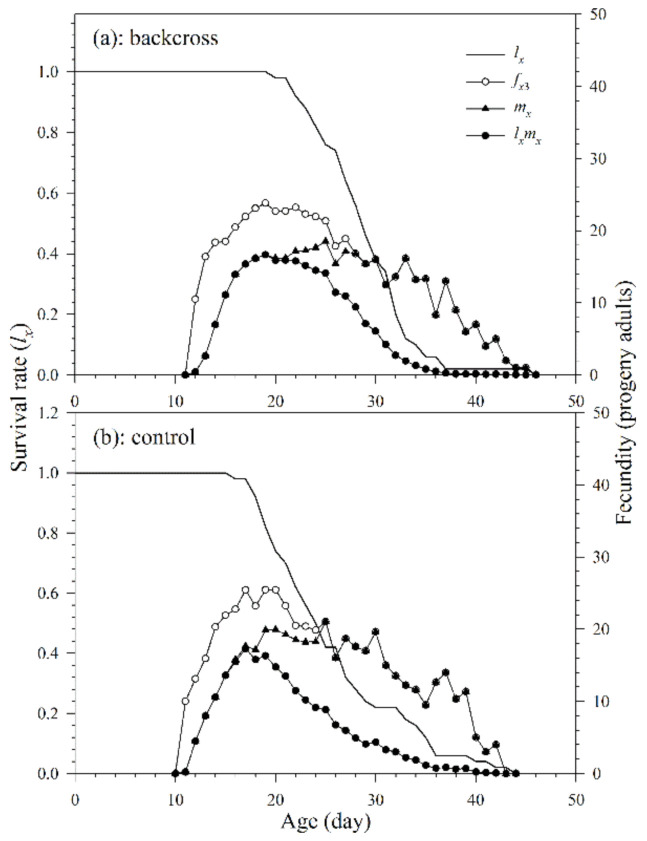
Age-specific survival rates (*l_x_*), age-stage specific fecundities (*f_x_*_3_), age-specific fecundities (*m_x_*), and age-specific net fecundities (*l_x_m_x_*) of *A. asychis* in the backcross treatment (**a**) and control (**b**).

The reproduction period in the backcross treatment and control was 16.7 and 15.2 d, respectively. In addition, there was no significant difference in the oviposition period (Table 3 and Figure 5). The age-specific survival rate (*l_x_*) is the probability that a new egg will survive to age *x* and is calculated by taking all surviving individuals of both sexes and those that died during pre-adult stages. The curve (*f_x_*_3,_ the number of parasitoid progeny reproduced by female adults at age *x* and stage *j*) shows age-stage specific fecundity of females in the third life stage. It shows that the peak reproductions of the parasitoids in the backcross treatment and control both occurred on the 19th day, with corresponding *f_x_*_3_ values of 23.8 progeny adults and 25.5 progeny adults, respectively. The curves of age-specific fecundities (*m_x_*) and age-specific net fecundities (*l_x_m_x_*) of the parasitoids of the backcross treatment show that the peak reproductions of the parasitoids occurred respectively on the 25th and 19th day (18.5 and 16.7 progeny adults, respectively). The curves of *m_x_* and *l_x_m_x_* of *A. asychis* adult females in the control group show that the peak reproductions occurred on the 25th (21.1 progeny adults) and 17th day (17.3 progeny adults), respectively. In addition, the *f_xj_*, *m_x_* and *l_x_m_x_* curves show rough peaks in reproduction, and these peaks may be due to *A. asychis*’s reproductive physiology.

#### 3.2.2. Population Growth Parameters

The *r* and *λ* of *A. asychis* in the backcross treatment were significantly lower than those in the control. However, the *R*_0_ and *T* in the backcross treatment were greater than those in the control, but this difference was only significant for *T* (Table 4).

#### 3.2.3. Host Feeding

**Figure 6:**
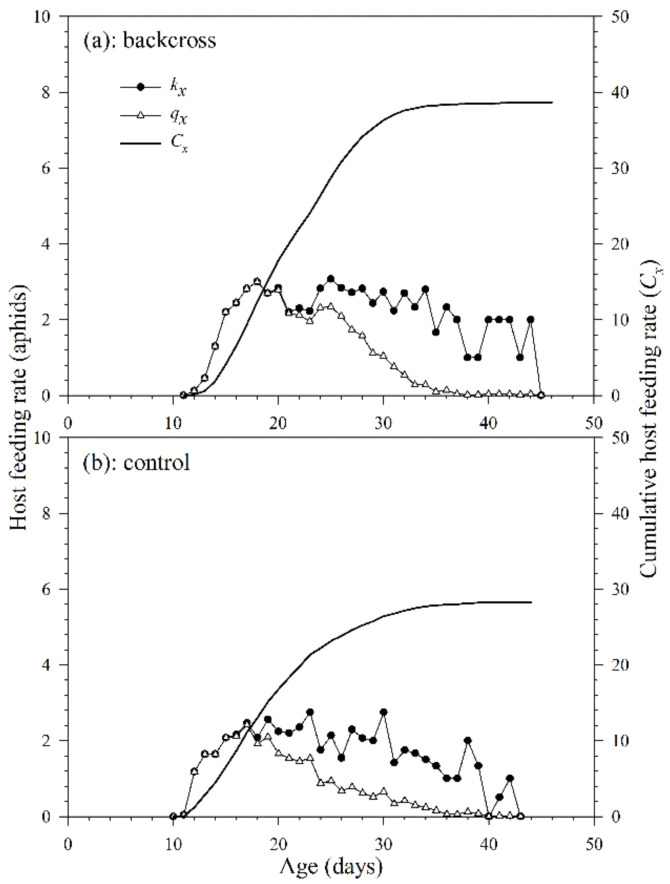
Age-specific host feeding rates (*k_x_*), age-specific net host feeding rates (*q_x_*), and cumulative host feeding rates (*C_x_*) of *A. asychis* in the backcross treatment (**a**) and control (**b**).

The age-specific host feeding rate (*k_x_*) of *A. asychis* in the backcross and control showed irregular peaks during the adult stage (Figure 6). The maximum daily age-specific host feeding rate (*k_x_*) in the backcross treatment (4.0 aphids at 41st day) was similar to that in the control (4.0 aphids at 40th day). The maximum daily age-specific net host feeding rates (*q_x_*) in the backcross treatment and the control were 3.0 aphids on the 18th day and 1.9 aphids on the 17th day. The cumulative host feeding rate (*cumu. C_x_*) is shown in Figure 6. The net host feeding rate (*C*_0_) of *A. asychis* in the backcross treatment was significantly higher than that in control following bootstrap analysis, and the values were 38.7 and 23.2 aphids, respectively. There were no significant differences in the stable host feeding rate (*ψ*) and finite host feeding rate (*ω*) of *A. asychis* between the backcross treatment and control (Table 4).

#### 3.2.4. Non-Effective Parasitism Rate

**Figure 7:**
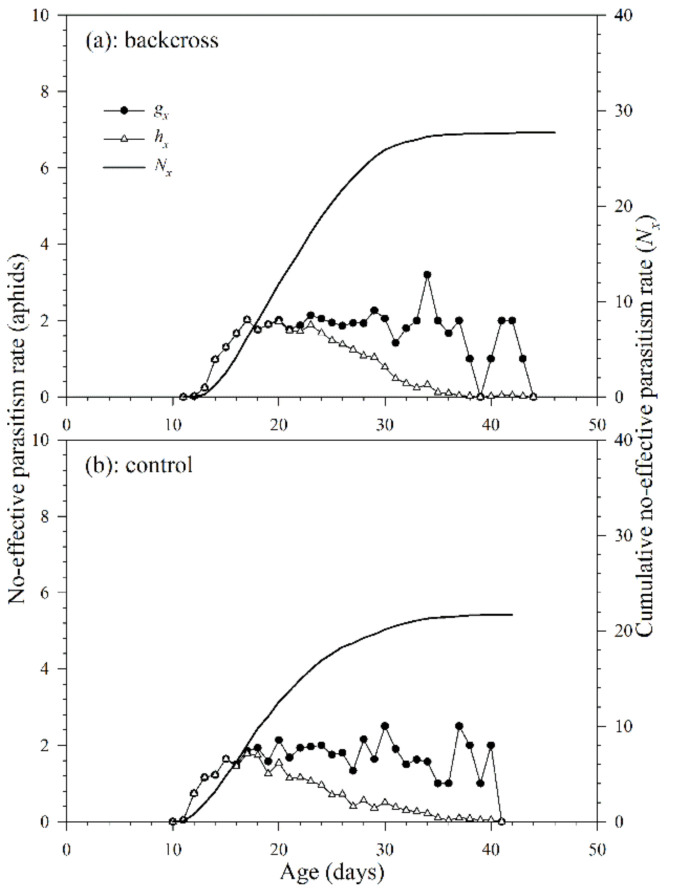
Age-specific non-effective parasitism rates (*g_x_*), age-specific net non-effective parasitism rates (*h_x_*) and cumulative non-effective parasitism rates (*N_x_*) of *A. asychis* in the backcross treatment (**a**) and control (**b**).

The non-effective parasitism rate is defined as the number of aphids that a parasitoid successfully parasitized, but no parasitoid adult emerged from the mummies. Because *A. asychis* could not parasitize aphids during the pre-adult stage, there was no noneffective parasitism rate before adulthood. The age-specific non-effective parasitism rate (*g_x_*) of *A. asychis* in the backcross treatment and control showed irregular peaks during the adult stage (Figure 7). The maximum values of age-specific net non-effective parasitism rates (*h_x_*) were 3.2 aphids on the 34th day in the backcross treatment and 2.8 aphids on the 30th day in controls. The maximum value of *h_x_* in the backcross treatment (2.0 aphids at 17th day) was greater than those in controls (2.4 aphids at 17th day). The age-specific non-effective parasitism rates (*g_x_*) at middle and late adult stages are higher than that in the early stage, indicating that the egg quality produced at the later stage may be lower than that in the early stage. In addition, as the age of *A. asychis* female adult increased, the ability of females to select suitable hosts might decrease, which might lead to an increase in the proportion of unsuitable hosts and further increases the age-specific non-effective parasitism rates (*g_x_*). The cumulative non-effective parasitism rate (*cumu. N_x_*) is shown in Figure 7. The net non-effective parasitism rate (*N*_0_) in the backcross treatment and control were 27.7 aphids and 28.2 aphids per female adult, respectively. The stable non-effective parasitism rate (*γ*) and finite non-effective parasitism rate (*ε*) of *A. asychis* in the backcross treatments were similar to those in the control group (Table 4).

#### 3.2.5. Killing Rate

**Figure 8:**
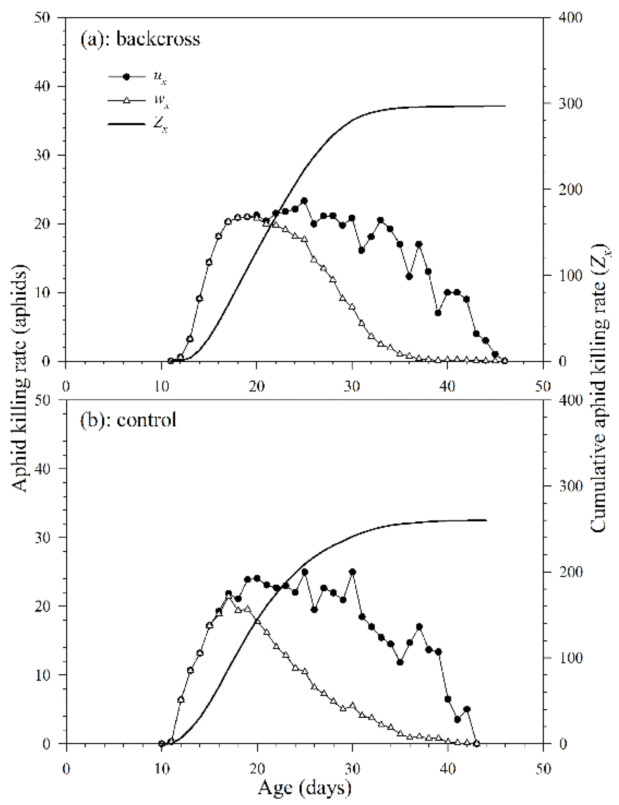
Age-specific killing rates (*u_x_*), age-specific net killing rates (*w_x_*) and cumulative killing rates (*Z_x_*) of *A. asychis* in the backcross treatment (**a**) and control (**b**).

The daily age-specific killing rate (*u_x_*) of *A. asychis* in the backcross treatment and control showed irregular peaks during the adult stage (Figure 8). The maximum values of *u_x_* in the backcross treatment and control were 25.1 aphids on the 30th day and 21.6 aphids on the 17th day, respectively. The age-specific net killing rates (*w_x_*) were 23.6 aphids on the 25th day in the backcross treatment and 20.9 aphids on the 18th day in control. The cumulative aphid killing rate (*cumu. Z_x_*) is shown in Figure 8. The net killing rates (*Z*_0_) of *A. asychis* in the backcross treatments were significantly greater than those in the control, and the values were 298.3 and 261.8 aphids, respectively. The stable killing rate (*θ*) and finite killing rate (*ϑ*) of *A. asychis* in the backcross treatments were similar to those in controls (Table 4). The transformation rate (*Q_p_*) of *A. asychis* in the control was significantly higher than those in the backcross treatment.

## 4. Discussion

In this study, we found that most mating in *A. asychis* involves four distinct stages: courtship, pre-copulatory, copulation, and post-copulatory behaviors. *A. asychis* females and males could mate at least five times. The total number of mummies, and both the female and male longevities, decreased significantly with an increase in mating frequency. On the contrary, the number of female progenies increased significantly with an increase of mating frequency. In addition, the intrinsic rate of increase (*r*) of *A. asychis* of the control group was significantly greater than that in the backcross treatment, while the finite killing rate (*θ*), i.e., control efficiency, of *A. asychis* in control was similar to that in the backcross treatment.

The results showed that most of both *A. asychis* female and male adults accepted multiple matings. Therefore, we speculated that female adults tended to accept multiple genotype sperms from different males to increase their genetic fitness. Multiple mating enables females to obtain sperm from different males and may be a better evolutionary reproductive strategy in *A. asychis* compared to other single-parasitic parasitoid species. For some species, not only sperm was transferred from male to female in the mating process, but also secretions containing bioactive molecules produced by the male’s accessory glands [31]. Such secretions may render her unwilling or unable to mate once again for some time [31]. The effects of secretions produced from the *A. asychis* male’s accessory gland on mating and female reproduction requires further investigation. In addition, the courtship duration of an *A. asychis* female that mated with the same male was longer than that that of one which mated with multiple males after the first mating, which suggested that there was individual recognition in *A. asychis,* and *A. asychis* female adults prefer to mate with new male adults than mated males.

In the multiple mating treatments, the longevity of *A. asychis* females and males reduced significantly as mating times increased. Similarly, *the longevity of Drosophila melanogaster* Meigen which had multiple mating was reported to be shorter than that which had a single mating [32]. The longevity of females and males of the diamondback moth, *Plutella xylostella* L., was significantly reduced as the mating times increased [33]. A similar relationship between female longevity and mating history was also found in many other insects, such as seed beetle *Callosobruchus maculates*, butterfly *Jalmenus evagoras*, male-dimorphic dung beetle *Onthophagus binodis,* parasitoid *Bathyplectes curculionis* and so on [34,35,36,37,38,39]. Therefore, we suggested that the negative effect of multiple mating on the insect longevity might be of reference significance for the study of lifespan in other higher animals and even humans.

Under complementary sex determination (CSD), females of Hymenoptera arise from diploid, fertilized eggs and males from haploid, unfertilized eggs [25]. For instance, only mated *A. asychis* females are able to produce female progenies, and unmated females only produce male progenies [1]. In our study, we noticed that with the increase in mating frequency, the percentage of *A. asychis* female progeny increased significantly, suggesting that the supply of sperm for *A. asychis* females that mated only once may be limiting. Perhaps most interestingly, the number of mummfied aphids decreased with an increase in mating frequency, while the number of female progenies increased as the mating frequency increased, which suggested that *A. asychis* females seem to decide how many fertilized eggs to oviposit based on the mating frequency and/or the number of sperm they obtain from males. A previous research reported that *A. asychis* females which mated on the 8th day and 15th day produced equal sex ratios after mating, but these were slightly more female-biased than the sex ratios of females which mated on the first day [40]. Together, those results suggests that the amount of sperm from one mating might be not enough to supply the female during its life span, and that sperm depletion might occur at the later stage of reproduction.

A common mating strategy in single-parasitic parasitoids is that male parasitoids can generally mate with multiple females but females mate only once [18]. For example, in the single-parasitic parasitoid *S. endius*, females generally mate only once in their life-span, although sperm might be exhausted in the later stage of reproduction [19,20,21]. In this study, we found that *A. asychis* females, single-parasitic parasitoid, mated multiple times significantly increased their proportion of female progenies, and the number of *A. asychis* female progenies produced by females with multiple mating were also significantly more than that produced by females which mated once, which indicates that *A. asychis* females might utilize the strategy of multiple matings to avoid the phenomenon of sperm exhaustion in the later stages of reproduction and further expand the population. This behavior indicates that *A. asychis* appear to have evolved a different mating strategy compared to other single-parasitic parasitoids.

In this study, we found that the total number of *M. persicae* nymphs killed by *A. asychis* females for obtaining nutrition varied irregularly, which was not affected by mating frequency. Over the short term, the number of mummified aphids decreased as mating frequency increased, which also means that the control efficiency decreased. However, the reproduction of more female progenies may be to serve as a supplement, to increase the F1 and subsequent generations, which in turn would eventually kill more pests. In addition, there is a contradiction for *A. asychis* females: when faced with an aphid host, how does it decide to feed or parasitize? According to our day-to-day observation, when the number of *M. persicae* nymphs was enough, very few parasitized aphid nymphs were killed by *A. asychis* female to obtain nutrition. On the other hand, when *M. persicae* nymphs were very severe, some parasitized *M. persicae* nymphs were killed by *A. asychis* females to obtain nutrition. An interesting observation were that no *A. asychis* females would parasitize the aphids that are eaten by the same or different *A. asychis* females. However, this judgment and identification mechanism is unclear now and should be researched in future.

The developmental time of *A. asychis* was significantly longer in the backcross treatment than that in the control, which suggested that the backcross might extend the developmental time of parasitoids. Some previous studies indicated that temperature, host species, and host age and size affect host suitability of *A. asychis*, especially developmental time [1,41,42,43]. Besides the host, the parasitoid strain can also affect the developmental time [44,45]. All these results showed that the developmental time of *A. asychis* may be affected by many biological and non-biological factors, such as temperature, backcrossing, geographic distribution, host species, age, and size.

We also found that the longevity of females and males in the backcross treatment was significantly longer than those in the controls, which showed that backcross had a prolonging effect on the longevity of *A. asychis* female and male progeny. The previous studies reported that the longevity of *A. asychis* was affected by many biological and abiotic factors, such as host plant, host age, temperature and so on. For example, the longevity of an *A. asychis* female reared on the *M. persicae* nymphs on chili pepper was significantly longer than when reared on the *M. persicae* nymphs on cabbage, the longevity of *A. asychis* male also showed the significant difference [4]. The mean longevity of *A. asychis* females when parasitizing and host feeding on 1–2 day old aphid nymphs was 32.8 days, while it was 25.2 and 24.2 days for host age group of 4–5 day old nymphs and adults, respectively [43]. The increase in temperature including 20, 24, 28, and 32 °C caused a significant decrease in the longevity of *A. asychis* females and males [46]. Given the complexity of the factors influencing the longevity of *A. asychis*, more investigation is needed.

In this study, the peak value of age-stage specific fecundities (*f_x_*_3_*)* of *A. asychis* in the backcross treatment was greater than those parasitizing 1–2 days old *Aphis gossypii*, but lower than those parasitizing second instar nymphs of *A. gossypii* [2,43]. The net reproductive rate (*R*_0_) of *A. asychis* in the backcross treatment was greater than those in the control group, but was lower than those with second instar nymphs of *A. gossypii* as host [2]. The differences in the fecundity of *A. asychis* might be due to different host species and parasitoid sources. In addition, the intrinsic rate of increase (*r*) and finite rate of increase (*λ*) of *A. asychis* in the backcross treatment was significantly lower than those in the control. This suggested that the capacity of increasing the population of *A. asychis* in the backcross treatment was lower than that in the control under ideal environmental conditions.

*Aphelinusasychis* females need to feed on hosts for nutrition. We found that the net host feeding rate (*N*_0_) in the backcross treatment was significantly greater than those in controls, indicating that the effect of backcross on the cumulative host feeding rate was positive. Tatsumi & Takada found that the number of *A. gossypii* killed by *A.* *asychis* female for host feeding was significantly greater than those of *M. persicae* or *Macrosiphum euphorbiae* Thomas [47]. Sengonca et al. also found that the number of *A. gossypii* killed by *A.* *asychis* female for host feeding decreased significantly as the host age increased [43]. Byeon et al. found that the total non-reproductive killing rate, i.e., host feeding or stinging, of *A.* *asychis* with 2nd and 3rd instar *A. gossypii* nymphs was significantly higher than those in this study [7]. These studies showed that *A.* *asychis* host feeding might be affected by many factors, including parasitoid source, host species and instars.

The age-specific non-effective parasitism rate (*g_x_*) showed irregular variations during the female’s life-span, and the values of *g_x_* at middle and later adult stages were higher than that at prophase. This phenomenon indicates that both female adult age and backcross might affect parasitism and progeny emergence. The net non-effective parasitism rate (*N*_0_) in the backcross treatment was higher than that in the control, which showed that the backcross negatively affected the reproduction of *A. asychis*.

Typically, the number of pests killed by the parasitoid in its lifespan represents individual control efficiency. Here, the net killing rate (*Z*_0_) in the backcross was similar to that in the control, implying that a significant difference in the control efficiency of *A. asychis* does not exist between backcross and control. However, the intrinsic rate of increase (*r*) of *A. asychis* in the control was significantly greater than that in the backcross treatment, which was opposite to the net killing rate. In a previous study, Chi et al. suggested the use of the finite predation rate to compare the control potential of different predators feeding on the same prey or of the same predator feeding on different prey [48]. Therefore, we used the finite killing rate to compare the control potential of *A. asychis* parasitizing *M. persicae* in the backcross treatment and control. The results showed that the finite killing rate in the control was greater than that in the backcross treatment, but was not significant. This demonstrated that the control efficiency of *A. asychis* on *M. persicae* in the control was nearly similar to the control efficiency in the backcross, when both the finite rate of increase (*λ*) and stable killing rate (*ϑ*) were considered.

## 5. Conclusions

The number and proportion of *A. asychis* female progenies increased significantly as the mating frequency increased. Therefore, increasing the released number of *A. asychis* male adults in biological control strategies, might be beneficial to increase the mating frequency of female adults and ultimately the control efficiency of the next generation. However, this requires further studies. Additionally, there was no significant difference in the finite killing rate of *A. asychis* between control and backcross treatment, which suggested that the backcross had a negligible negative effect on the control efficiency of the second generation of *A. asychis*. Even if all individuals released as biological control agents were females, backcross would ensure the continuity of the population. This means that the proportion of *A. asychis* females could be increased when needed and may result in a higher proportion of male progenies, which might lead to backcross. Therefore, the effect of higher female proportions on the continuous control efficiency and the appropriate proportion of female adults to be used in this biological control strategy under field conditions require further evaluation.

## Figures and Tables

**Table 1:** Population parameter definitions and formulae used in the computer programs including TWOSEX–MSChart and CONSUME–MSChart.

Parameter	Definition	Formula
*s_xj_*	Age-stage-specific survival rate	$s_{xj}=\frac{n_{xj}}{s_{01}}$
*l_x_*	Age-specific survival rate	$l_{x}=\sum_{j=1}^{\beta }s_{xj}$
*m_x_*	Age-specific fecundity	$m_{x}=\frac{\sum_{j=1}^{\beta }s_{xj}f_{xj}}{\sum_{j=1}^{\beta }s_{xj}}$
*r*	Intrinsic rate of increase	$\lambda =e^{r}$
*R*_0_	Net reproductive rate	$R_{0}=\overset{\infty }{\underset{x=0}{\sum }}l_{x}m_{x}$
*T*	Mean generation time	$T=\frac{\ln\left(R_{0}\right)}{r}$
*k_x_*	Age-specific host feeding rate	$k_{x}=\frac{\sum_{j=1}^{\beta }s_{xj}c_{xj}}{\sum_{j=1}^{\beta }s_{xj}}$
*q_x_*	Age-specific net host feeding rate	$q_{x}=l_{x}k_{x}$
*C*_0_	Net host feeding rate	$C_{0}=\overset{\infty }{\underset{x=0}{\sum }}l_{x}k_{x}$
*ψ*	Stable host feeding rate	$\psi =\overset{\infty }{\underset{x=0}{\sum }}\overset{\beta }{\underset{j=1}{\sum }}a_{xj}c_{xj}$
*ω*	Finite host feeding rate	ω=λφ
*g_x_*	Age-specific non-effective parasitism rate	$g_{x}=\frac{\sum_{j=1}^{\beta }s_{xj}d_{xj}}{\sum_{j=1}^{\beta }s_{xj}}$
*h_x_*	Age-specific net non-effective parasitism rate	$h_{x}=l_{x}g_{x}$
*N_0_*	Net non-effective parasitism rate	$N_{0}=\overset{\infty }{\underset{x=0}{\sum }}l_{x}g_{x}$
*γ*	Stable non-effective parasitism rate	$\gamma =\overset{\infty }{\underset{x=0}{\sum }}\overset{\beta }{\underset{j=1}{\sum }}a_{xj}d_{xj}$
*ε*	Finite non-effective parasitism rate	ε=λγ
*u_x_*	Age-specific aphid killing rate	$u_{x}=\frac{\sum_{j=1}^{\beta }s_{xj}p_{xj}}{\sum_{j=1}^{\beta }s_{xj}}$
*w_x_*	Age-specific net aphid killing rate	$w_{x}=l_{x}u_{x}$
*Z*_0_	Net aphid killing rate	$Z_{0}=\overset{\infty }{\underset{x=0}{\sum }}l_{x}u_{x}=R_{0}+C_{0}+N_{0}$
*ϑ*	Stable aphid killing rate	$\vartheta =\overset{\infty }{\underset{x=0}{\sum }}\overset{\beta }{\underset{j=1}{\sum }}a_{xj}p_{xj}$
*θ*	Finite aphid killing rate	θ=λϑ
*Q_p_*	Transformation rate	$Q_{p}=\frac{Z_{0}}{R_{0}}=\frac{R_{0}+C_{0}+N_{0}}{R_{0}}$

The *n_xj_* was the survival number of parasitoid at age *x* and stage *j*. The age-stage-specific fecundity (*f_xj_*) was the number of parasitoid progeny at age *x* and stage *j*. The age-stage-specific host feeding rate (*c_xj_*) was the number of aphid nymphs killed by *A. asychis* female adults at age *x* and stage *j* for feeding. The age-stage-specific non-effective parasitism rate (*d_xj_*) was the number of aphid nymphs parasitized by *A. asychis* at age *x* and stage *j* but for which emergence failed. The age-stage-specific aphid killing rate (*p_xj_*) was the number of aphid nymphs fed on by *A. asychis* at age *x* and stage *j*, and the *p_xj_* value is the sum of *f_xj_, c_xj_*, and *d_xj_*. In addition, the intrinsic rate of increase (*r*) is the maximum rate of population growth. The finite predation rate (*θ*) was used to compare the control potential of different parasitoids parasitizing the same host or of the same parasitoids parasitizing different hosts.

**Table 2:** Duration in seconds of four mating stages in the mating process of the same or different *A. asychis* males.

Mating Times	1	2	3	4	5	df	*F*	*p*
Same male—Courtship	61.3 ± 5.4 A	162.2 ± 9.9 B *	192.6 ± 13.0 B *	280.2 ± 29.0 C *	464.4 ± 24.9 D *	4128	76.509	<0.001
Different males—Courtship	59.3 ± 3.4 a	66.8 ± 3.0 ab	90.2 ±6.5 bc	109.5 ± 8.4 c	233.5 ± 22.8 d	4128	60.195	<0.001
df	78	69	58	42	25			
*F*	3.449	9.527	7.350	6.331	6.819			
*p*	0.135	<0.001	<0.001	<0.001	<0.001			
Same male—Precopulatory	63.1 ± 3.2 A	64.1 ± 3.5 A	75.8 ± 3.6 A	64.5 ± 6.2 A	72.8 ± 5.0 A	4128	2.080	0.087
Different males—Precopulatory	61.7 ± 3.2 a	59.0 ± 2.9 a	67.2 ± 3.0 a	66.6 ± 3.8 a	68.2 ± 3.7 a	4128	1.651	0.165
df	78	69	58	42	25			
t	0.313	1.132	4.021	0.303	0.756			
*p*	0.755	0.262	0.653	0.764	0.457			
Same male—Copulation	6.9 ± 0.6 A	7.1 ± 0.6 A	8.1 ± 0.8 A	7.8 ± 1.1 A	5.5 ± 0.5 A	4128	1.119	0.350
Different males—Copulation	6.5 ± 0.5 a	6.7 ± 0.7 a	6.8 ± 0.8 a	6.8 ± 0.9 a	6.7 ± 0.8 a	4128	0.047	0.996
df	78	69	57	42	25			
*F*	0.46	0.404	1.054	0.684	1.292			
*p*	0.647	0.688	0.296	0.497	0.208			
Same male—Post copulatory	181.4 ± 7.8 A	171.5 ± 10.1 A	199.3 ± 11.8 A	179.3 ± 9.3 A	183.9 ± 13.0 A	4103	1.005	0.408
Different males—Post copulatory	166.3 ± 6.2 a	179.9 ± 7.4 a	178.3 ± 5.6 a	183.3 ± 7.6 a	178.2 ± 8.7 a	4122	1.015	0.402
df	67	57	45	33	23			
*F*	1.518	0.685	1.752	0.333	0.37			
*p*	0.134	0.496	0.087	0.741	0.715			

Different capital cases represent significant difference in the same females which mated with the same single male among all mating frequencies. Different lowercase represent significant differences in the same females which mated with different males among all mating frequencies. * represents a significant difference in mating between the same or different males at the same mating frequency.

**Table 3:** Developmental time and offspring of *A. asychis* which fed on 2nd instar *M. persicae* nymphs in the backcross treatment and control.

Stage	Backcross		Control		*t*	*p*
	*n*	Mean ± SE	*n*	Mean ± SE		
Egg + larva (d)	50	7.8 ± 0.2 a	50	6.4 ± 0.1 b	24.571	<0.001
Pupa (d)	50	6.7 ± 0.2 a	50	6.3 ± 0.1 b	19.284	0.003
Preadult (d)	50	14.5 ± 0.2 a	50	12.7 ± 0.2 b	26.335	<0.001
Female longevity (d)	35	31.1 ± 0.7 a	34	28.5 ± 1.2 b	28.214	<0.001
Female adult longevity (d)	35	16.7 ± 0.7 a	34	15.5 ± 1.1 a	2.137	0.162
Male longevity (d)	15	24.7 ± 0.6 a	16	20.6 ± 0.7 b	14.262	0.005
Male adult longevity (d)	15	9.9 ± 0.7 a	16	8.6 ± 0.7 a	2.864	0.157
Reproduction period (d)	35	16.7 ± 0.7 a	34	15.2 ± 1.1 a	1.935	0.219
Progeny (egg/female)	35	331.3 ± 13.6 a	34	309.4 ± 21.8 a	2.094	0.131

Female longevity means the whole lifespan of from egg to adult death, and female adult longevity means the duration of life from emergence until death. Means in the same row followed by different letters denote significant differences between backcross and control treatments according to *t*-test at 5% significant level based on bootstrap technique. The standard errors were calculated using the bootstrap procedure with 100,000 bootstraps.

**Table 4:** Population parameters, host feeding rate, non-effective parasitism rate, and killing rate of *A. asychis* parasitizing *M. persicae* in the backcross treatment and control.

Parameters	Backcross	Control	*t*	*p*
Intrinsic rate of increase, *r* (d^−1^)	0.2687 ± 0.0060 a	0.2858 ± 0.0070 b	29.672	<0.001
Finite rate of increase, *λ* (d^−1^)	1.3082 ± 0.0078 a	1.3308 ± 0.0093 b	23.251	0.002
Net reproductive rate, *R*_0_ (offspring)	231.9 ± 23.4 a	210.4 ± 25.2 a	1.567	0.232
Mean generation time, *T* (d)	20.3 ± 0.3 a	18.7 ± 0.3 b	28.232	<0.001
Net host feeding rate, *C*_0_ (aphids)	38.7 ± 4.0 a	23.2 ± 3.0 b	35.817	<0.001
Stable host feeding rate, *ψ*	0.0549 ± 0.0050 a	0.0397 ± 0.0045 b	42.890	<0.001
Finite host feeding rate, *ω*	0.0718 ± 0.0069 a	0.0528 ± 0.0063 b	33.795	<0.001
Net non-effective parasitism rate, *N*_0_ (aphids)	27.7 ± 2.9 a	28.2 ± 3.3 a	2.134	0.195
Stable non-effective parasitism rate, *ε*	0.0366 ± 0.0033 a	0.0540 ± 0.0053 b	24.059	0.006
Finite non-effective parasitism rate, *γ*	0.0478 ± 0.0046 a	0.0719 ± 0.0075 b	25.770	<0.001
Net killing rate, *Z*_0_ (aphids)	298.3 ± 30.0 a	261.8 ± 31.1 a	2.184	0.178
Stable killing rate, *ϑ*	0.3999 ± 0.0332 a	0.4251 ± 0.0399 a	1.319	0.224
Finite killing rate, *θ*	0.5232 ± 0.0464 a	0.5657 ± 0.0570 a	2.953	0.194
Transformation rate, *Q_p_*	1.2864 ± 0.0068 a	1.2443 ± 0.0059 b	27.033	<0.001

Means in the same row followed by different letters denote significant differences between backcross and control treatments according to *t*-test at 5% significant level based on bootstrap technique. The standard errors were calculated using the bootstrap procedure with 100,000 bootstraps.

## Data Availability

Not applicable.

## References

[1] Raney H.G., Coles L.W., Eikenbary R.D., Morrison R.D., Starks K.J. (1971). Host preference, longevity, developmental period and sex ratio of *Aphelinus asychis* with 3 sorghum-fed species of aphids held at controlled temperatures. Ann. Entomol. Soc. Am..

[2] Byeon Y.W., Tuda M., Takagi M., Kim J.H., Choi M.Y. (2011). Life history parameters and temperature requirements for development of an aphid parasitoid *Aphelinus asychis* (Hymenoptera: Aphelinidae). Environ. Entomol..

[3] Byeon Y.W., Tuda M., Kimb J.H., Choi M.Y. (2011). Functional responses of aphid parasitoids, *Aphidius colemani* (Hymenoptera: Braconidae) and *Aphelinus asychis* (Hymenoptera: Aphelinidae). Biocontrol Sci. Technol..

[4] Wang S.Y., Chi H., Liu T.X. (2016). Demography and parasitic effectiveness of *Aphelinus asychis* reared from *Sitobion avenae* as a biological control agent of *Myzus persicae* reared on chili pepper and cabbage. Biol. Control.

[5] Wang S.Y., Liang N.N., Tang R., Liu Y.H., Liu T.X. (2016). Brief heat stress negatively affects the population fitness and host feeding of *Aphelinus asychis* (Hymenoptera: Aphelinidae) parasitizing *Myzus persicae* (Hemiptera: Aphididae). Environ. Entomol..

[6] Wang S.Y., Zhang D.Y., Liu T.X. (2020). Influence of temperature on the demographics and control efficiency of *Aphelinus asychis*, a parasitoid of the cabbage pest, *Myzus persicae*. Phytoparasitica.

[7] Byeon Y.W., Tuda M., Takagi M., Kim J.H., Kim Y.H. (2009). Non-reproductive host killing caused by *Aphelinus asychis* (Hymenoptera: Aphelinidae), a parasitoid of cotton aphid, *Aphis gossypii* (Homoptera: Aphididae). J. Fac. Agric. Kyushu Univ..

[8] Logan J.A., Wollkind D.J., Hoyt S.C., Tanigoshi L.K. (1976). An analytic model for description of temperature dependent rate phenomena in arthropods. Environ. Entomol..

[9] Zhang S.Z., Cao Z., Zhang F., Liu T.X. (2014). Exposing eggs to high temperatures affects the development, survival and reproduction of *Harmonia axyridis*. J. Thermal Biol..

[10] Geden C.J., Kaufman P.E. (2007). Development of *Spalangia cameroni* and *Muscidifurax raptor* (Hymenoptera: Pteromalidae) on live house fly (Diptera: Muscidae) pupae and pupae killed by heat shock, irradiation, and cold. Environ. Entomol..

[11] Roux O., Lann C.L., van Alphen J.J.M., van Baaren J. (2010). How does heat shock affect the life history traits of adults and progeny of the aphid parasitoid *Aphidius avenae* (Hymenoptera: Aphidiidae). Bull. Entomol. Res..

[12] Firake D.M., Khan M.A. (2014). Alternating temperatures affect the performance of *Trichogramma* species. J. Insect Sci..

[13] El-Hafez A.A., El-Sharkawy M.A.A., Karim A.H. (2014). Consequential effects of high temperature on biological characteristics influencing the efficacy of *Trichogramma evanescens* Westwood and its progeny. Egypt J. Biol. Pest Control.

[14] Wang M.X., Cui S.M., Wang H.B., Li Z.X., Li H.T., Zhang X., Hu B., Zhang X.B., Zhang X.M. (2008). The performance of illumination, temperature and humidity in solar greenhouse. Greenh. Hortic..

[15] Ribaut J.M., Ragot M. (2007). Marker-assisted selection to improve drought adaptation in maize: The backcross approach, perspectives, limitations, and alternatives. J. Exp. Bot..

[16] Reynolds J.D. (1996). Animal breeding systems. Trends Ecol. Evol..

[17] Arnqvist G., Nilsson T. (2000). The evolution of polyandry: Multiple mating and female fitness in insects. Anim. Behav..

[18] Ridley M. (1993). Clutch size and mating frequency in parasitic hymenoptera. Am. Nat..

[19] King B.H., Bressac C. (2010). No fitness consequence of experimentally induced polyandry in a monandrous wasp. Behaviour.

[20] King B.H., Fischer C.R. (2010). Male mating history: Effects on female sexual responsiveness and reproductive success in the parasitoid wasp *Spalangia endius*. Behav. Ecol. Sociobiol..

[21] King B.H., Saporito K.B., Ellison J.H., Bratzke R.M. (2005). Unattractiveness of mated females to males in the parasitoid wasp *Spalangia endius*. Behav. Ecol. Sociobiol..

[22] Damiens D., Boivin G. (2005). Male reproductive strategy in *Trichogramma evanescens*: Sperm production and allocation to females. Physiol. Entomol..

[23] Charnov E.L. (1982). Parent-offspring conflict over reproductive effort. Am. Nat..

[24] Godfray H.C.J. (1994). Parasitoids: Behavioral and Evolutionary Ecology.

[25] Cournault L., Aron S. (2009). Diploid males, diploid sperm production, and triploid females in the ant *Tapinoma erraticum*. Naturwissenschaften.

[26] Li C.D., Byeon Y.W., Choi B.R. (2007). An Aphelinid species, *Aphelinus asychis* Walker (Hymenoptera: Aphelinidae) new to Korea. J, Asia-Pac. Entomol..

[27] Chi H. (2017). TWOSEX-MSChart: Computer Program for Age Stage, Two-Sex Life Table Analysis. http://140.120.197.173/ecology/.

[28] Chi H. (2017). CONSUME-MSChart: Computer Program for Consumption Rate Analysis Based on the Age Stage, Two-Sex Life Table. http://140.120.197.173/ecology/.

[29] Efron B., Tibshirani R.J. (1993). An Introduction to the Bootstrap.

[30] Akköprü E., Atlıhan R., Okut H., Chi H. (2015). Demographic assessment of plant cultivar resistance to insect pests: A case study of the dusky-veined walnut aphid (Hemiptera: Callaphididae) on five walnut cultivars. J. Econ. Entomol..

[31] Gillott C. (2003). Male accessory gland secretions: Modulators of female reproductive physiology and behavior. Annu. Rev. Entomol..

[32] Chapman T., Liddle L.F., Kalb J.M., Wolfner M.F., Partridge L. (1995). Cost of mating in *Drosophila melanogaster* females is mediated by male accessory gland products. Nature.

[33] Wang X.P., Fang Y.L., Zhang Z.N. (2005). Effect of male and female multiple mating on the fecundity, fertility, and longevity of diamondback moth, *Plutella xylostella* (L.). J. Appl. Entomol..

[34] Rutowski R.L. (1982). Mate choice and Lepidoptera mating behavior. Fla. Entomol..

[35] Savalli U.M., Fox C.W. (1999). The effect of male mating history on paternal investment, fecundity and female remating in the seed beetle *Callosobruchus maculates*. Funct. Ecol..

[36] Haughes L., Chang B.S.W., Wangner D., Pierce N.E. (2000). Effects of mating history on ejaculate size, fecundity, longevity, and copulation duration in the ant-tended lycaenid butterfly, *Jalmenus evagoras*. Behav. Ecol. Sociobiol..

[37] Kotiaho J.S., Simmons L.W. (2003). Longevity cost of reproduction for males but no longevity cost of mating or courtship for females in the male-dimorphic dung beetle *Onthophagus binodis*. J. Insect Physiol..

[38] Paukku S., Kotiaho J.S. (2005). Cost of reproduction in *Callosobruchus maculatus*: Effects of mating on male longevity and the effect of male mating status on female longevity. J. Insect Physiol..

[39] Jacob H.S., Evans E.W. (2000). Influence of carbohydrate foods and mating on longevity of the parasitoid *Bathyplectes curculionis* (Hymenoptera: Ichneumonidae). Environ. Entomol..

[40] Fauvergue K., Hopper K.R., Antolin M.F., Kazmer D.J. (1998). Does time until mating affect progeny sex ratio? A manipulative experiment with the parasitoid wasp *Aphelinus asychis*. J. Evolution. Biol..

[41] Jackson H.B., Eikenbary R.D. (1971). Bionomics of *Aphelinus asychis* (Hymenoptera: Eulophidae) an introduced parasite of the sorghum greenbug. Ann. Entomol. Soc. Am..

[42] Kang E.J., Byeon Y.W., Kim J.H., Choi M.Y., Choi Y.S. (2012). The effect of temperatures on the biological characteristics of two aphid parasitoids *Aphelinusasychis* (Walker) and *Aphelinus varipes* (Forster) (Hymenoptera: Aphelinidae) on two aphid hosts. Korean J. Appl. Entomol..

[43] Sengonca C., Schirmer S., Blaeser P. (2008). Life table of the aphid parasitoid *Aphelinus asychis* (Walker) (Hymenoptera, Aphelinidae) parasitizing different age groups of *Aphis gossypii* Glover (Homoptera, Aphididae). J. Plant Dis. Protect..

[44] Bernal J., Gonzalez D. (1993). Temperature requirements of four parasites of the Russian wheat aphid *Diuraphis noxia*. Entomol. Exp. Appl..

[45] Lee J.H., Elliott N.C. (1998). Comparison of developmental responses to temperature in *Aphelinus asychis* (Walker) from two different geographic regions. Southwest. Entomol..

[46] Wang S.Y., Wang L.B., Yan G.L., Liu Y.H., Zhang D.Y., Liu T.X. (2020). Temperature-dependent demographic characteristics and control potential of *Aphelinus asychis* reared from *Sitobion avenae* as a biological control agent for *Myzus persicae* on chili peppers. Insects.

[47] Tatsumi E., Takada H. (2005). Evaluation of *Aphelinus asychis* and *Aphelinus albipodus* (Hym., Aphelinidae) as biological control agents against three pest aphids. Appl. Entomol. Zool..

[48] Chi H., Mou D.F., Allahyari H., Yu J.Z., Huang Y.B., Yang T.C., Farhadi R., Gholizadeh M. Finite predation rate: A novel parameter for the quantitative measurement of predation potential of predator at population level. Nat. Preced..

